# Anxiety and depression in school-age patients with spinal muscular atrophy: a cross-sectional study

**DOI:** 10.1186/s13023-021-02008-8

**Published:** 2021-09-09

**Authors:** Mei Yao, Yu Xia, Yijie Feng, Ying Ma, Yi Hong, Yanyi Zhang, Jie Chen, Changzheng Yuan, Shanshan Mao

**Affiliations:** 1 Department of Neurology, Children’s Hospital, Zhejiang University School of Medicine, National Clinical Research Center for Child Health, Hangzhou, 310052 China; 2 Department of Psychology, The Children’s Hospital, Zhejiang University School of Medicine, National Clinical Research Center for Child Health, Hangzhou, China; 3 Centre for Global Health, Zhejiang University School of Medicine, Hangzhou, 310052 China; 4 School Public Health of Zhejiang University, The Children’s Hospital, Zhejiang University School of Medicine, National Clinical Research Center for Child Health, Hangzhou, 310052 China

**Keywords:** Depression, Anxiety, Spinal muscular atrophy, School-age, SCARED, DSRSC

## Abstract

**Background:**

Spinal muscular atrophy (SMA) is a rare neurogenetic disease which involves multisystem dysfunctions such as respiratory, digestive, and motor disorders. Anxiety, depression and other psychological disorders often accompany severe chronic physical diseases. The aim of this study was to investigate the prevalence of anxiety and depression along with their influencing factors among school-age patients with SMA.

**Methods:**

We conducted a cross-sectional study on school-age SMA patients in China. Patients aged 8–18 years with a genetic diagnosis of 5qSMA were invited to answer a questionnaire composed of sociodemographic and clinical questions, then to complete the Screen for Child Anxiety-Related Emotional Disorders and Depression Self-Rating Scale for depression and anxiety level evaluation. At the end of the questionnaire, further questions assessed the subjective anxiety and subjective depression of patients’ caregivers and their expectations for their child’s future.

**Results:**

Complete data were available for 155 patients. The sample included 45.8% boys and 54.2% girls; 65.2% were type II, 27.1% were type III, and the remainder were type I SMA. Rates of anxiety and depression in these school-age SMA patients were 40.0% and 25.2%, respectively. Gender, age, and disease type were not associated with anxiety or depression, but respiratory system dysfunction, digestive system dysfunction, skeletal deformity, rehabilitation exercise, academic delay, specialized support from school, household income level, caregivers’ subjective anxiety, and caregivers’ expectations were significantly related to both anxiety and depression.

**Conclusions:**

There was a high prevalence of anxiety and depression in school-age SMA patients in China. Professional psychological care maybe included in the standard of care. These results also call for possible targets for intervention such as reducing complications, improving drug accessibility, retaining normal schooling, strengthening school support, and enhancing the ability of the caregivers of SMA patients to assist in the diagnosis and treatment of the disease, so improving the mental health of SMA patients.

## Background

Spinal muscular atrophy (SMA) is a rare, autosomal-recessive neuromuscular disorder caused by genetic mutation in the 5q13 survival of motor neuron (SMN) 1 gene [1]. This mutation results in reduced levels of the SMN protein, causing muscle weakness and atrophy [2]. With progress of the disease, SMA often involves multisystem physical dysfunction such as respiratory, digestive, cardiovascular, and motor disorders, which seriously reduce the patients’ quality of life [3, 4].

Somatic diseases play an extremely important role in the occurrence and development of mental disorders. Numerous studies have shown that patients with somatic diseases—including asthma, allergic rhinitis, stroke, multiple sclerosis, inflammatory bowel disease, leukemia, cancer, and other chronic diseases—frequently suffer from anxiety and depression [5–9]. Anxiety is the most common mental health disorder in childhood; it is also among the earliest psychiatric conditions to manifest, at a median age of 11 years [10]. General anxiety prevalence rates are 5.7% to 12% among children under 18 years [11]. The World Health Organization (WHO) identified depression and depressive disorders as “priority mental health disorders of adolescence” due to their high rates of prevalence and recurrence, and the significant complications and impairment that can occur [12].

It is widely known that anxiety and depression have a substantial influence on physical diseases, complicating disease symptoms and increasing the difficulty of diagnosis and treatment. Many studies have shown that anxiety and depressive disorders can aggravate the symptoms of physical diseases and adversely affect prognosis through neuroendocrine and immune mechanisms [13, 14]. Patients with depression and anxiety often show decreased interest in social activities, lack of motivation, and impaired cognitive function, which further reduce their social functioning and quality of life. It is reported that patients with somatic diseases combined with anxiety have moderate to severe social functional deficits [15]. In addition, studies have repeatedly emphasized that depression is an important risk factor for memory impairment and dementia, which can reduce cognitive function, while severe anxiety or depression can lead to suicidal behavior [16].

Faced with the multisystem dysfunction characteristic of SMA, the functional activities of patients are limited and they often suffered from a variety of complications, hence the impact of SMA on mental health can be severe [17]. A series of 25 interviews conducted with SMA adults in Australia highlighted the challenges in mental health that need to be met [18]. Mazzella et al. found that SMA patients reported their mental health, including anxiety and depression, to be significantly affected by disease [19].

The psychological health of school-age patients with SMA has not been systematically analyzed: it is essential to closely monitor for psychological disorders, especially anxiety and depression, in these patients. The purpose of the current study was to describe the prevalence of anxiety and depression and to investigate the risk factors for these in school-age SMA patients.

## Methods

### Study design and recruitment

**Fig. 1:**
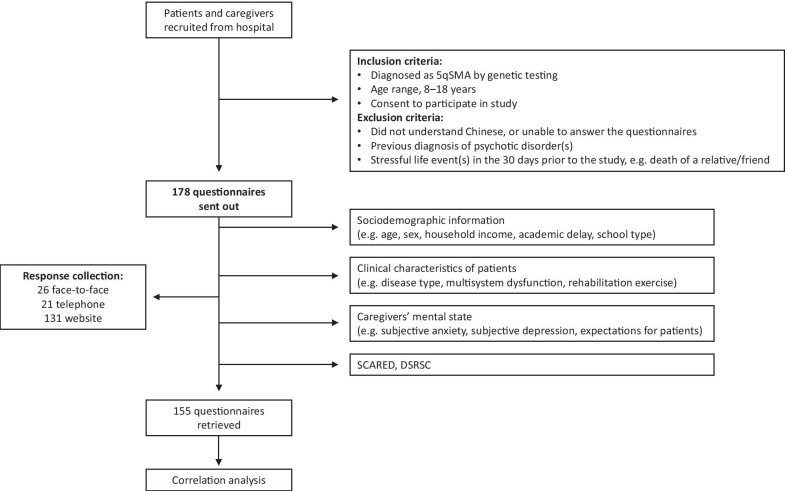
Study flow chart

A prospective, observational study was conducted from January 1st to February 15th, 2021. The participants included school-age SMA patients and their caregivers recruited from the Department of Neurology of the Children’s Hospital of Zhejiang University School of Medicine in Hangzhou, China. The inclusion criteria for patients were: (1) diagnosis of 5qSMA by genetic testing, (2) age range 8–18 years, and (3) consent to participate in the study. Exclusion criteria included participants who did not understand Chinese or were unable to answer the questionnaire (reading problems); previous diagnosis of psychotic disorders; or stressful life events in the 30 days prior to the study, such as death of a relative/friend, personal accident, or accident affecting someone close to them. The study questionnaire was completed by face-to-face or telephone interview, or via an online platform (Fig. 1). Fulfilling the principles of voluntary participation, participants were provided with detailed information about the purpose and methods of this study, and all patients or their legally authorized representatives provided written informed consent. The study was approved by the Ethics Committee of the Children’s Hospital of Zhejiang University School of Medicine (2019-IRB-171).

### Potentially associated factors

Patient age, gender, and disease type were considered as potentially associated factors. Besides, support from the school and teachers and academic delay are factors to consider. School type was classified into two categories depending on the support from the school and teachers: (1) personalized school is regarded as which one can obtain special academic/emotional/physical support from the school or teachers. For example, personalized schools are equipped with escalators which can reduce the difficulty of climbing stairs. Toilets seats have also been deemed as the personalized support that it was more comfortable and convenient than squat toilets. (2) Traditional school means a mainstream school with no supporting program for SMA patients, lacking personalized support in regards of personal and equipment. Academic delay was defined as the child who is age-appropriate to participate in grade enrollment but not catching up the school as usual. For example, most children will begin the (first) grade in primary school in China when they are 7 years old. If a child attends the (first) grade in primary school beyond 7 years old, we consider this situation as an academic delay. Regarding the financial aspects of care, annual household income level was separated into two categories with the China Statistical Yearbook of the data in 2019: high (≥ 200,000 RMB) and low (< 200,000 RMB). Clinical multisystem dysfunction was included as a factor in our study, comprising respiratory system dysfunction, digestive system dysfunction, and skeletal deformity. Respiratory function dysfunction was defined as abnormal lung ventilation or ventilation due to a variety of causes, including repeating cough, expectation, prolonged pneumonia, dyspnea, etc. The definition of skeletal dysfunctions was structural changes in the skeletal system that result in functional changes. The main symptoms of skeletal dysfunctions included scoliosis, tendon contracture, joint deformation, hip dislocation, etc. Digestive system dysfunctions were defined as disordered functions of digestive organs including the gastrointestinal tract, the biliary and liver system, and the pancreas, such as salivation, difficulty swallowing, constipation, reflux and delayed gastric emptying, etc. In addition, patients were divided into a rehabilitation exercise group and non-rehabilitation training group, depending on they performed rehabilitation exercise or not. Empathy—i.e. the experience and understanding of others’ feelings and emotions while recognizing these as distinct from our own—can serve as both a personality disposition and a psychological process [20]. For this reason, we included the caregivers’ subjective anxiety, subjective depression, and expectations for patients as possible influencing factors, and explored the correlation between these and the prevalence of anxiety and depression in patients. We have independently designed 3 questions, that is, whether parents have anxiety or depression for their children's current disease status in the past 1 month, and whether they have expectations for their children's treatment (excluding other stress events, such as the loss of a spouse, car accident, etc.). The answer to these questions just needs a simple yes or no to measure caregivers' subjective mental states.

### Anxiety and depression measures

The Screen for Child Anxiety-Related Emotional Disorders (SCARED) and the Depression Self-Rating Scale for Children (DSRSC) were used to assess the patients’ anxiety and depression in the study.

The SCARED is a self-report tool created by Birmaher to screen for anxiety disorders in children [21]. There are 41 items in five subscales: somatic/panic disorder, generalized anxiety disorder, separation anxiety disorder, social phobia, and school phobia. Each item is scored on a three-point Likert scale of 0 (not true, or hardly ever true), 1 (sometimes true), or 2 (true or often true). The cut-off score for the SCARED is 25, and higher scores indicate greater anxiety severity. Based on SCARED scores, the patients were divided into two groups: anxious (score ≥ 25) and non-anxious (score < 25). Acceptable reliability and validity have been established for this scale in China with Cronbach’s alpha ranges from 0.43 to 0.89 [22].

The DSRSC is a self-report questionnaire designed to evaluate depression in children. It consists of 18 items and each of the items are scored on a 3-point scale (0 = never, 1 = sometimes, 2 = most) [23]. This test has a maximum score of 54. After completing the questionnaire, patients were categorized into two groups based on their DSRSC score: depressive (score range, 15–54) and non-depressive (score range, 0–14). In China, the DSRSC has high reliability and validity, and Cronbach’s alpha is 0.72 [24].

### Statistical analysis

SPSS version 24.0 (IBM) was used for statistical analyses. Continuous variables are presented as means ± standard deviations (SD), and categorical variables are presented as numbers (percentages). A chi-squared test was utilized to test for significance across major subgroups. Spearman correlation coefficient was calculated to evaluate the correlations of socio-demographics and clinical characteristics with anxiety and depression. A *p*-value < 0.05 was considered significant.

## Results

### Patient characteristics

A total of 178 questionnaires were sent out and 155 were retrieved, with an effective rate of 87.0%. Complete data were available for the 155 patients whose score was calculable and fit the inclusion criteria. Patients in the study had an age range of 8–18 years, with a mean age of 10.28 ± 2.98 years; most patients (78.7%, *n* = 122) were 8–12 years old. Seventy-one (45.8%) patients were male and 84 (54.2%) were female. Over half of the patients interviewed reported that they had academic delay. Only 48 patients were in personal school while others were in traditional school. There were only 8 patients who were treated with Nusinursen, and 3 of them reached the diagnosis of anxiety or depression.

There were 60 (40.0%) patients with a SCARED score ≥ 25, and fit the definition of being anxious in our study. Moreover, 39 (25.2%) patients had a DSRSC score ≥ 15, meeting the diagnostic criteria for depression. Sociodemographic characteristics, clinical characteristics, and psychosocial aspects of the patients in our study are summarized in Table 1. Additionally, most caregivers responded “Yes” to questions on their subjective anxiety (83.9%) and subjective depression (80.0%). The caregivers’ subjective mental evaluation is described in Table 2.

**Table 1:** The sociodemographic and clinical characteristics of SMA patients

Variable	Description
*Gender*	
Male	71 (45.8)
Female	84 (54.2)
*Age subgroups*	
8–12 years	122 (78.7)
13–18 years	33 (21.3)
Age (mean ± SD)	10.28 ± 2.98 years
*Types of SMA disease*	
Type I	12 (7.7)
Type II	101 (65.2)
Type III	42 (27.1)
*Respiratory system dysfunction*	
Yes	118 (76.1)
No	37 (23.9)
*Digestive system dysfunction*	
Yes	133 (85.8)
No	22 (14.2)
*Skeletal deformity*	
Yes	55 (35.5)
No	100 (64.5)
*Rehabilitation exercise*	
Yes	83 (53.5)
No	72 (46.5)
*Academic delay*	
Yes	85 (54.8)
No	70 (45.2)
*Type of school*	
Traditional	107 (69)
Personalized	48 (31)
*Household income*	
High (≥ 200,000 RMB)	23 (14.8)
Low (< 200,000 RMB)	132 (85.2)
*SCARED score*	
≥ 25	62 (40.0)
< 25	93 (60.0)
*DSRSC score*	
≥ 15	39 (25.2)
< 15	116 (74.8)

All values are *n* (%) except where otherwise indicated

**Table 2:** The characteristics of caregivers of SMA patients

Variables	*n* (%)
*Caregivers’ subjective anxiety*	
Yes	130 (83.9)
No	25 (16.1)
*Caregivers’ subjective depression*	
Yes	124 (80.0)
No	31 (20.0)
*Caregivers’ expectations*	
Yes	114 (73.5)
No	41 (26.5)

### Correlation of anxiety with sociodemographic and clinical characteristics in SMA patients

The prevalence of anxiety was similar in males and females (r =  − 0.016, *p* = 0.843) and did not differ significantly according to age (r = 0.058, *p* = 0.471) or disease type (r = 0.046, *p* = 0.829). With regard to the clinical characteristics of SMA patients, respiratory system dysfunction (r = 0.253, *p* = 0.002), digestive system dysfunction (r = 0.196, *p* = 0.015), and skeletal deformity (r = 0.193, *p* = 0.016) were significantly correlated with anxiety. The findings also showed a significantly lower prevalence of severe anxiety among patients performing rehabilitation exercise compared with those not doing any exercise (r =  − 0.180, *p* = 0.025). Additionally, patients with academic delay were more susceptible to anxiety than those without. Regarding the household income level, the findings showed that patients with a high annual household income (≥ 200,000 RMB) had a significantly lower prevalence of depression compared with patients from low-income families. The Spearman correlation coefficient test showed that a significant positive correlation existed between anxiety and academic delay (r = 0.185, *p* = 0.021) while a significant inverse correlation existed between anxiety and household income level (r =  − 0.193, *p* = 0.012) (Table 3).

**Table 3:** Correlation of anxiety with sociodemographic and clinical characteristics

Variables	Anxiety	Non-anxiety	*r*	*P*
*Gender*			− 0.016	0.843
Male	29 (40.8%)	42 (59.2%)		
Female	33 (39.3%)	51 (60.7%)		
*Age subgroups*			0.058	0.471
8–12 years	47 (38.5%)	75 (61.5%)		
13–18 years	15 (45.5%)	18 (54.5%)		
*Types of SMA disease*			0.046	0.829
Type I	4 (33.3%)	8 (66.7%)		
Type II	40 (39.6%)	61 (60.4%)		
Type III	18 (42.9%)	24 (57.1%)		
*Respiratory system dysfunction*			0.253	**0.002**
Yes	23 (62.2%)	14 (37.8%)		
No	39 (33.1%)	79 (66.9%)		
*Digestive system dysfunction*			0.196	**0.015**
Yes	14 (63.6%)	8 (36.4%)		
No	48 (36.1%)	85 (63.9%)		
*Skeletal deformity*			0.193	**0.016**
Yes	47 (47.0%)	53 (53.0%)		
No	15 (27.3%)	40 (72.7%)		
*Rehabilitation exercise*			− 0.180	**0.025**
Yes	22 (30.6%)	50 (69.4%)		
No	40 (48.2%)	43 (51.8%)		
*Academic delay*			0.185	**0.021**
Yes	27 (31.8%)	58 (68.2%)		
No	35 (50.0%)	35 (50.0%)		
*Type of school*			− 0.262	**0.001**
Personalized	10 (20.8%)	38 (79.2%)		
Traditional	52 (48.6%)	55 (51.4%)		
*Household income*			− 0.193	**0.012**
High (≥ 200,000 RMB)	4 (17.4%)	19 (82.6%)		
Low (< 200,000 RMB)	58 (43.9%)	74 (56.1%)		

Bold indicates *p*-values under 0.05

As detailed in Table 4, the Spearman correlation coefficient test indicated that patients’ anxiety was positively correlated with caregivers’ subjective anxiety (r = 0.358, *p* < 0.001). Furthermore, caregivers' subjective depression also had a significant association with patients’ anxiety, such that the patients’ anxiety increased as their caregivers’ subjective depression increased (r = 0.342, *p* < 0.001). Additionally, the prevalence of anxiety in patients was lower when caregivers had high expectations than when caregivers had low expectations, a difference that was statistically significant (r =  − 0.167, *p* = 0.037).

**Table 4:** Correlation of anxiety with caregivers’ mental state

Variables	Anxiety	Non-anxiety	*r*	*P*
*Caregivers’ subjective anxiety*			0.358	** < 0.001**
Yes	62 (47.7%)	68 (52.3%)		
No	0 (0.0%)	25 (100.0%)		
*Caregivers’ subjective depression*			0.342	** < 0.001**
Yes	60 (48.4%)	64 (51.6%)		
No	2 (6.5%)	29 (93.5%)		
*Caregivers’ expectations*			− 0.167	**0.037**
Yes	40 (35.1%)	74 (64.9%)		
No	22 (53.7%)	19 (46.3%)		

Bold indicates *p*-values under 0.05

### Correlation of depression with sociodemographic and clinical characteristics in SMA patients

With regards to the correlation between gender and depression, male patients did not differ statistically from female patients (r =  − 0.064, *p* = 0.428). Besides, there was no correlation between the type of SMA and depression in our study (r =  − 0.111, *p* = 0.114). The results of respiratory system evaluation revealed that patients with respiratory system dysfunction had a higher prevalence of depression than those without respiratory system dysfunction (r = 0.268, *p* = 0.001). Similarly, the prevalence of depression in patients with digestive system dysfunction (r = 0.318, *p* =  < 0.001) or skeletal deformity (r = 0.181, *p* = 0.024) was significantly different than in those without these factors. The findings also showed the prevalence of depression in patients with rehabilitation exercise was significantly lower than in those without rehabilitation exercise (r =  − 0.182, *p* = 0.023). With respect to the existence or nonexistence of academic delay, the results indicated that the patients with academic delay had a significantly higher prevalence of anxiety than those without academic delay (r = 0.280, *p* < 0.001). There was also a significant correlation between school type and depression (r =  − 0.195, *p* < 0.015). As distinct from anxiety, the prevalence of depression in patients was not significantly different between high-income and low-income families (r = 0.009, *p* = 0.912) (Table 5).

**Table 5:** Correlation of depression with sociodemographic and clinical characteristics

Variables	Depression	Non-depression	*r*	*P*
*Gender*			− 0.064	0.428
Male	20 (28.2%)	51 (71.8%)		
Female	19 (22.6%)	65 (77.4%)		
*Age subgroups*			0.134	0.095
8–12 years	27 (22.1%)	95 (77.9%)		
13–18 years	12 (36.4%)	21 (63.6%)		
*Types of SMA disease*			− 0.111	0.114
Type I	6 (50.0%)	6 (50.0%)		
Type II	24 (23.8%)	77 (76.2%)		
Type III	9 (21.4%)	33 (78.6%)		
*Respiratory system dysfunction*			0.268	**0.001**
Yes	17 (45.9%)	20 (54.1%)		
No	22 (18.6%)	96 (81.4%)		
*Digestive system dysfunction*			0.318	** < 0.001**
Yes	13 (59.1%)	9 (40.9%)		
No	26 (19.5%)	107 (80.5%)		
*Skeletal deformity*			0.181	**0.024**
Yes	31 (31.0%)	69 (69.0%)		
No	8 (14.5%)	47 (85.5%)		
*Rehabilitation exercise*			− 0.182	**0.023**
Yes	12 (16.7%)	60 (83.3%)		
No	27 (32.5%)	56 (67.5%)		
*Academic delay*			0.280	** < 0.001**
Yes	12 (14.1%)	73 (85.9%)		
No	27 (38.6%)	43 (61.4%)		
*Type of school*			− 0.195	**0.015**
Personalized	6 (12.5%)	42 (87.5%)		
Traditional	33 (30.8%)	74 (69.2%)		
*Household income*			0.009	0.912
High (≥ 200,000 RMB)	6 (26.1%)	17 (73.9%)		
Low (< 200,000 RMB)	33 (25.0%)	99 (75.0%)		

Bold indicates *p*-values under 0.05

Additionally, the results showed that the patients’ depression level was positively correlated with the caregivers' subjective anxiety (r = 0.173, *p* = 0.018). Within groups, the prevalence of depression in patients was not significantly different according to the caregivers’ subjective depression (r = 0.067, *p* = 0.405). In addition, the results showed that caregivers’ expectations significantly affected the prevalence of depression in patients (r =  − 0.158, *p* = 0.049) (Table 6).

**Table 6:** Correlation of depression with caregivers’ mental state

Variables	Depression	Non-depression	*r*	*P*
*Caregivers’ subjective anxiety*			0.173	**0.018**
Yes	37 (28.5%)	93 (71.5%)		
No	2 (8.0%)	23 (92.0%)		
*Caregivers’ subjective depression*			0.067	0.405
Yes	33 (26.6%)	91 (73.4%)		
No	6 (19.4%)	25 (80.6%)		
*Caregivers’ expectations*			− 0.158	**0.049**
Yes	24 (21.1%)	90 (78.9%)		
No	15 (36.6%)	26 (63.4%)		

Bold indicates *p*-values under 0.05

## Discussion

The current study reports firstly on the prevalence rates and risk factors for anxiety and depression among school-age patients with SMA in China. Anxiety and depression were observed in 40.0% and 25.2% of SMA patients, respectively, in our study. We also found that the prevalence of depression and anxiety in SMA patients differed significantly across school type, academic delay, household economic level, clinical characteristics of disease, caregivers’ mental health, and caregivers’ expectations for patients.

Our findings indicated that the prevalence of anxiety and depression in school-age SMA patients were not significantly different between female and male children with SMA. Whether gender has a differential influence on the prevalence of anxiety and depression remains controversial. The WHO reported that depression is more common among females (5.1%) than males (3.6%). As with depression, anxiety disorders are more common among females than males [12]. Linden et al. identified a large gender gap in depression and anxiety among patients with cancer, where the prevalence in women was two to three times higher than seen for men [8]. In a Canadian study, Marrie et al. found that women experienced a higher level of depression in multiple sclerosis [25]. Females also had significantly higher rates of anxiety than males among children and adolescents with cerebral palsy [26]. In contrast to these findings, a study comparing women and men with chronic urticaria patient found no significant differences in total anxiety and depression scores [27]. Inconsistent conclusions about the relationship between gender and anxiety and depression may be explained by the different incidence of diseases between genders. The lack of a significant difference in the prevalence of anxiety and depression between genders in our study might be explained by the fact that SMA morbidity is unrelated to gender.

In the present study, patients with type III and type I SMA had the highest and the lowest prevalence rates of anxiety, respectively, although this difference did not attain statistical significance. By contrast, the highest and lowest prevalence rates of depression were found in type I and type III patients, respectively, also with no statistically significant difference. A possible explanation for these observations is that type III patients retain more mobility than type I patients and can therefore maintain normal contacts, but that type III patients cannot enjoy the same activity levels as their normal peers, which leads to anxiety. On the other hand, type I patients are more likely to suffer from depression because of their limited mobility and therefore inability to socialize normally. Our results also indicated that the prevalence rates of depression and anxiety in patients with SMA showed no significant difference with age, although anxiety and depression occurred more frequently in adolescent patients aged 13–18 years. These results are consistent with to the WHO report that anxiety and depression are more likely to occur in older than younger people.

A scoping systematic review of adults with SMA concluded that greater psychosocial wellbeing was associated with poorer physical function [18]. Our research found that patients with respiratory dysfunction had significantly higher anxiety and depression rates than patients without respiratory dysfunction. Given the association between psychological distress and poorer respiratory outcomes, this result is in agreement with studies on other respiratory diseases, previously reported. For example, one study found that 50% and 39% of patients with chronic obstructive pulmonary disease showed clinically significant anxiety and depression, respectively [28]. Moreover, a nationwide population-based survey in Korea reported that allergic rhinitis and rhinosinusitis synergistically compromise the mental health and health-related quality of life of patients [29]. Patients with asthma also have a high prevalence of depression, and we can hypothesize that the long-term course of the disease in SMA increases the patients’ anxious and depressive symptoms [30].

SMA patients often have digestive system complications such as constipation and gastroesophageal reflux. In the current study, the patients with digestive system dysfunction had a significantly higher anxiety and depression rate than those without digestive system complications. Studies have found that constipation and diarrhea often aggravate the anxiety of patients, and anxiety may affect the course of the disease through the neuroendocrine regulatory system, further aggravating the clinical symptoms of patients and exacerbating constipation [31, 32]. A study on Parkinson’s disease (PD) patients showed that severe constipation and motor symptoms were closely related to depression; the researchers explained that involvement of the enteric plexus of the gastrointestinal tract and dorsal motor nucleus of the vagus (DMV) initially causes constipation in PD patients, and subsequent spread to the raphe nuclei and locus coeruleus leads to depressive mood [33]. Furthermore, gastroesophageal reflux reduces the quality of life of patients, so that the long-term course of disease promotes an increase in psychological pressure and damage to mental health, leading to anxiety and depression [6].

Skeletal deformities can lead to motor dysfunction in patients. As the disease progresses, the majority of SMA patients experience skeletal system dysfunctions such as hip joint dislocation, wrist joint deformity, ankle joint deformity, scoliosis, etc., leading to claudication, limitation in mobility, and reduced outdoor activities [34, 35]. Our study found that patients with skeletal deformities had significantly higher rates of anxiety and depression than those without skeletal deformities, which is in agreement with the results of psychological surveys on other musculoskeletal disorders. For example, the anxiety score in fracture patients was significantly higher than in healthy people [36]. An investigation of depression rates in patients with hereditary paraplegia showed that their anxiety rate reached the survey 58% of the population is considered to be related to the patient’s mobility level [37]. Pedras et al. also found a high prevalence of anxiety and depression symptoms among patients after a lower limb amputation [38]. A further study suggested that patients with rheumatoid arthritis (RA) tend to experience anxiety, because RA is a chronic disease that causes pain, stiffness, swelling, and limitations in the motion and function of multiple joints [39]. Ying Qian et al. also found SMA patients always experienced heartbreak, fear, embarrassed and frustrated with loss of functional abilities or a physical disability [40]. All of the evidence demonstrates the psychological aspects of SMA should be highly valued.

The collective evidence shows that patients with motor functional disorders are more likely to have anxiety and depression. Psychological factors can also affect the treatment of physical diseases as it may decrease the treatment compliance (i.e. rehabilitation) in patients. Skeletal deformity affects the patient’s motor function, reduces quality of life, and increases anxiety. Further, skeletal deformities are often accompanied by changes in body shape and abnormal walking posture, affecting appearance and causing pain and anxiety for the patient. According to our research, we suggest that patients with skeletal deformities should undergo corrective surgery or wear orthoses to correct or prevent the deformity from worsening. Patients without skeletal deformities should be managed as early as possible, through attending regular physician appointments to properly maintain reasonable rehabilitation exercises.

SMA is a neuromuscular degenerative disease characterized by progressive muscle atrophy and muscle weakness. Progressive motor regression is the most notable characteristic of the disease. Rehabilitation exercise, as one of the most important treatment strategies, can significantly improve the condition of the disease and the quality of life of patients [41]. Our results show that the anxiety rate of SMA patients who perform rehabilitation exercise is significantly lower than in those without rehabilitation training. The possible explanation for this difference is that rehabilitation exercise can improve patients' motor function and quality of life to a certain extent, thereby reducing the incidence of patients' anxiety and depression. At present, there is no detailed research to explain how exercise relieves anxiety and depression, but it is hypothesized that the mechanism of action is similar to that of antidepressants, with effects including increased expression of brain-derived neurotrophic factor (BDNF), increased availability of serotonin and norepinephrine, regulation of hypothalamic–pituitary–adrenal axis activity, and reduced systemic inflammatory signaling [42]. Additionally, Schuch et al. contend that physical activity can confer protection against the emergence of anxiety. In particular, higher physical activity levels protect against agoraphobia and post-traumatic stress disorder [43]. Therefore, reinforcing rehabilitation exercise is not only conducive to improving patients’ physical motor function, but may also support mental health by reducing the occurrence of anxiety and depression.

School education is very important to the physical and mental development of children. Education consists not only in teaching academic subjects but also in cultivating other skills in children, especially in morality, social interaction, and gradually improving their own personality. Many children with SMA cannot participate normally in school, leading to academic delay. Our study found that the anxiety and depression rates of children with academic delay were significantly higher than in those without academic delay. We further assessed the association between academic delay and anxiety and depression by adjusting covariates such as household income level and caregivers’ mental state, statistical significance remained (anxiety OR 2.23, 95%CI: 1.109 to 4.867; *p* = 0.025; depression OR 3.696, 95%CI: 1.556 to 7.855; *p* = 0.002, data not shown), which suggests that academic delay is an independent risk factor for the prevalence of anxiety and depression in SMA patients. This is probably because academic delay further limits social interaction, in addition with the loss of benefits from school, leading to increased psychological stress, anxiety, and depression. Moreover, anxiety and depression rates of SMA patients varied significantly between different types of schooling, with lower rates among patients enrolled in personalized schools compared with those attending traditional schools. Jacobsen et al. found that school type has an important impact on cognitive stimulation, when private schools provide more personalized methods [44]. One involved patients, caregivers and clinicians of SMA survey found patients were frustrated by lack of handicapped access or limited ability to socialize because of weakness and fatigue [40]. The reason for the significant differences in anxiety and depression in our study may be that personalized schools provide special support for SMA children, such as special desks and suitable classroom locations and recovery equipment. Therefore, in response to this situation, we encourage children with SMA to participate in school education, and call for schools to provide reasonable campus support, to optimize learning programs, and thereby to reduce the incidence of anxiety and depression in SMA patients.

Economic income determines the level of medical services and the quality of treatment for patients. We therefore investigated the prevalence rates of anxiety and depression in SMA patients with different household income levels. The results showed that the anxiety and depression rates of patients in high-income families were significantly lower than those in low-income families. Our results provide similar conclusions to studies on the prevalence of anxiety and depression in multiple sclerosis patients, which showed that patients with good economic status had the lowest levels of depression and anxiety [45, 46]. In addition, despite the continuous improvement in country and social support are gradually increasing in China, drug treatment for SMA is still not included in medical insurance which indicates the treatment needs of SMA patients has not been met yet. Hence, we conclude that economic status is one of the most important factors of anxiety and depression in school-age SMA patients. Economic support for the diagnosis and treatment of SMA patients needs to be greatly expanded in China, in addition to developing medical policies based on the capacity of SMA families to pay, reducing the economic burden on patients, improving the quality of nursing, and lowering the incidence of anxiety and depression.

Daily self-management refers to the ability to plan and arrange one's daily life, to control oneself and to deal with interpersonal relations. SMA patients have poor self-management ability and need caregivers to take care of daily life over an extended period. As we all know, emotional resonance is a psycho-social phenomenon, and personal mood is usually affected by the people surrounding us. Therefore, our research investigated the mental state of SMA caregivers and their expectations for disease treatment. The results showed that the mental state of caregivers can have a significant impact on anxiety and depression in SMA patients. A positive correlation was found between caregivers’ anxiety or depression and patients’ anxiety or depression, except there was no relation between caregivers’ depression and patients’ depression. In addition, our study found that caregivers’ expectations for diagnosis and treatment of SMA patients can significantly affect patients’ anxiety and depression. Anxiety and depression rates were significantly lower in caregivers with high expectations than in those with low expectations. Considering that caregivers have certain self-judgment and emotional control capabilities, it can be expected that guiding and resolving the caregiver’s psychology to reduce their subjective feelings of anxiety and depression may indirectly improve the anxiety and depression in SMA patients. Hence, it is important to encourage caregivers to maintain a positive attitude towards life and increase their confidence in the treatment of patients, which can reduce the possibility of anxiety and depression in the patients.

The consensus statement for standard of care (SOC) in SMA promotes multidisciplinary approaches, including pulmonary care, gastrointestinal and nutritional care, and orthopedic care and rehabilitation, which are helpful to improve SMA patients’ clinical symptoms, delay disease progression, and prolong survival [47]. It is remarkable, however, that psychological care is not included in the SOC of SMA, as previous studies have shown that SMA patients always suffer from mental problems. Our study also demonstrates that there are high prevalence rates of anxiety and depression in SMA patients of school age. Together, these findings highlight the need for the SOC in SMA to include professional psychological support.

A major strength of our study was, first, to measure the prevalence rates of anxiety and depression in SMA patients. There are currently very few articles studying the psychology of SMA patients, and no systematic reports on the evaluation of anxiety and depression in children with SMA. Our study therefore represents an advance in assessing the anxiety and depression state of SMA patients. In addition, our research analyzes the factors that may affect the mental state of children with SMA, including the patient’s sociodemographic and clinical characteristics, and analyzes the influence of the caregiver’s subjective mental state and expectations on the anxiety and depression of SMA patients. Our findings emphasize the importance of increased focus on the psychological aspects of SMA care, including the indirect regulation of the psychological state of SMA patients by influencing the outlook of caregivers.

The present study also has several limitations. First, the low sample size could limit study precision. The small number of patients also affects the subgroup analyses for many of the potential moderators, which should be considered in light of a lack of statistical power to draw definitive conclusions. Currently Nusinursen is the only available treatment drug on the market in China due to the differences in the medical and health care systems and most patients can't afford it—it's too expensive in China. We can’t make definite conclusions regarding the Nusinursen treatment because of the small number of sample size. Future research needs to be based upon much larger samples in drug treatment domains to certify whether Nusinursen or Risdiplam treatment is an important factor in reducing the incidence of anxiety and depression in patients. Our study was also cross-sectional, so it was not possible to determine the cause and effect relationship between the variables. Thirdly, the evaluation scales used are universal tools for assessing the mental state of children. At present, there is no psychological evaluation scale dedicated to SMA disease.

Further research is warranted using a larger survey sample at the individual level. In the meantime, psychological intervention measures to treat anxiety and depression require exploration in further follow-up observations. Finally, more research should be devoted to the development of a psychological scale dedicated to SMA disease.

## Conclusions

In this study sample of SMA patients in China, depression and anxiety were prevalent, respectively affecting over one-quarter and one-third of the study population. These mental health disorder symptoms were associated with respiratory system dysfunction, digestive system dysfunction, skeletal deformity, rehabilitation exercise, household income level, school type, caregivers’ subjective anxiety or depression, as well as caregivers’ expectations. Professional psychological care for SMA patients is essential. Scaling-up of mental health services—including personalized schooling, enhancing the mental health and expectations of caregivers, and precise management of multisystem disorders—is also crucial for the prevention of anxiety and depression in patients with SMA. Establishing the support on escalators and toilet seats to be used in school for improving ability of self-care, setting up a study group consisting of 5 to 7 teachers to assist learning and the use of buddy system of classmates to support the SMA children, etc. Poor access to drugs is still an important factor hindering the treatment of SMA in China. The advocacy of government funding for the available but expensive treatment and reducing the economic burden for the SMA patients is important. It is urgent to for the care and drugs of SMA to be covered by the healthcare system. Through disease awareness and public education, psychological health of caregivers can be improved. Which in turn can raise the quality of care for children with SMA, standardize treatment, and have regular multidisciplinary long-term follow-up management. Such efforts aim to improve the quality of life of SMA patients, helping them to achieve their full life potential.

## Data Availability

The datasets used and analysed during the current study are available from the corresponding author on reasonable request.

## Abbreviations

BDNF: Brain-derived neurotrophic factor
CI: Confidence interval
COPD: Chronic obstructive pulmonary disease
DMV: Dorsal motor nucleus of the vagus
DSRSC: Depression self-rating scale for children
HPA: Hypothalamic-pituitary-adrenal
OR: Odds ratio
PD: Parkinson’s disease
RA: Rheumatoid arthritis
SCARED: Screen for child anxiety-related emotional disorders
SD: Standard deviation
SMA: Spinal muscular atrophy
*SMN1*: Survival of motor neuron 1 (gene)
SOC: Standard of care
WHO: World Health Organization
